# Thioredoxin Confers Intrinsic Resistance to Cytostatic Drugs in Human Glioma Cells

**DOI:** 10.3390/ijms19102874

**Published:** 2018-09-21

**Authors:** Bodo Haas, Lena Schütte, Maria Wos-Maganga, Sandra Weickhardt, Marco Timmer, Niels Eckstein

**Affiliations:** 1 Federal Institute for Drugs and Medical Devices, Kurt-Georg-Kiesinger-Allee 3, 53175 Bonn, Germany; lenaschuette@gmx.net (L.S.); maria.wos-maganga@bfarm.de (M.W.-M.); sandra.weickhardt@bfarm.de (S.W.); 2 Faculty of Applied Natural Sciences, Cologne University of Applied Sciences, Kaiser-Wilhelm-Allee, 51368 Leverkusen, Germany; 3 Department of General Neurosurgery, Center for Neurosurgery, University Hospital Cologne, 50937 Cologne, Germany; marco.timmer@uk-koeln.de; 4 Applied Pharmacy, University of Applied Sciences Kaiserslautern, Campus Pirmasens, Carl-Schurz-Str. 10-16, 66953 Pirmasens, Germany; niels.eckstein@hs-kl.de

**Keywords:** glioblastoma, drug resistance, thioredoxin, TXNIP, PX-12

## Abstract

Thioredoxin (Trx) overexpression is known to be a cause of chemotherapy resistance in various tumor entities. However, Trx effects on resistance are complex and depend strictly on tissue type. In the present study, we analyzed the impact of the Trx system on intrinsic chemoresistance of human glioblastoma multiforme (GBM) cells to cytostatic drugs. Resistance of GBM cell lines and primary cells to drugs and signaling inhibitors was assessed by 3-(4,5-dimethylthiazol-2-yl)-2,5-diphenyltetrazolium bromide (MTT) assays. Impact of Trx inhibition on apoptosis was investigated by proteome profiling of a subset of proteins and annexin V apoptosis assays. Trx-interacting protein (TXNIP) was overexpressed by transfection and protein expression was determined by immunoblotting. Pharmacological inhibition of Trx by 1-methyl-2-imidazolyl-disulfide (PX-12) reduced viability of three GBM cell lines, induced expression of active caspase-3, and reduced phosphorylation of AKT-kinase and expression of β-catenin. Sensitivity to cisplatin could be restored by both PX-12 and recombinant expression of the upstream Trx inhibitor TXNIP, respectively. In addition, PX-12 also sensitized primary human GBM cells to temozolomide. Combined inhibition of Trx and the phosphatidylinositide 3-kinase (PI3K) pathway resulted in massive cell death. We conclude that the Trx system and the PI3K pathway act as a sequential cascade and could potentially present a new drug target.

## 1. Introduction

Gliomas are the most common primary brain tumors in humans, with glioblastoma multiforme (GBM, WHO grade IV) as the most aggressive form with the highest incidence [1,2]. GBM is treated with adjuvant radiotherapy and chemotherapy after surgery, but still, the median survival is only 14.6 months and the 5-year survival rate is 6% under therapy. Actually, the only chemotherapy regime resulting in a significant benefit in overall survival is adjuvant temozolomide (TMZ) following radiotherapy [3,4,5]. GBM therapy is aggravated by eventual development of intrinsic resistance of the tumor to radio- and chemotherapy. Resistance is frequently driven by mutated tumor suppressor genes after loss of function like *P53*, retinoblastoma protein (*RB*), and phosphatase and tensin homolog (*PTEN*) but also alterations in upstream receptor tyrosine kinase (RTK) signaling pathways [6,7,8].

Thioredoxin (Trx) is a 12 kDa ubiquitous protein of 104 amino acids with reducing activity. Trx is involved in cellular redox homeostasis, maintaining the balance between reactive oxygen species (ROS) generation and elimination [9]. Its active site comprises two cysteine residues which serve as a general disulfide oxidoreductase. Trx-interacting protein (TXNIP), a 46 kDa tumor suppressor protein, is also known as vitamin-D_3_-upregulated protein 1 (VDUP1) [10]. TXNIP is an upstream, negative regulator of the Trx-reducing activity. This functional antagonism comprises both suppression of Trx expression and direct protein interaction. Like other thiol-containing proteins, Trx overexpression has been suspected as a cause of chemotherapy resistance [11]. Hence, Trx overexpression in several tumor-derived cell lines is associated with resistance to cisplatin [12]. However, Trx effects on anticancer drug resistance are complex and depend strictly on the tissue type. For instance, cisplatin resistance in bladder and prostate cancer cell lines is triggered by Trx overexpression [13]. In contrast, cisplatin resistance in ovarian cancer cell lines is associated with high Trx levels, but recombinant Trx overexpression in nonresistant cells does not confer resistance to cisplatin [14]. Interestingly, it has been demonstrated that low TXNIP expression in glioma is associated with higher histopathological glioma grade and shorter overall patient survival. Furthermore, knockdown of TXNIP in the U251 GBM cell line promoted cell invasion, migration, and proliferation [15]. In this context, it is interesting to note that Trx expression correlates with glioma grade [16] and high Trx levels in breast cancer are associated with poor patient survival [17].

Another important player in the regulation of Trx function is Trx reductase (TrxR), which reduces oxidized disulfide-containing Trx back to the dithiol form. Inhibition of TrxR by specific inhibitors leads to an increase in oxidized, nonfunctional Trx. Consequently, targeting the Trx system, either by inhibition of Trx or TrxR, has been proposed as a promising strategy for cancer treatment [18,19]. This is reflected in anticancer clinical trials currently ongoing with the TrxR inhibitor auranofin (NCT03456700, NCT01737502, NCT01747798, NCT02770378). Auranofin is an organic gold complex clinically used as antiarthritic drug. In various cancer cells, auranofin has been shown to elicit strong cytotoxicity through diverse mechanisms such as overproduction of ROS, inhibition of glycolysis and the proteasome, and activation of apoptotic pathways [20,21,22,23,24,25,26]. Furthermore, auranofin treatment has been described to overcome cisplatin resistance in ovarian cancer cells [27].

There is currently one ongoing clinical trial combining TMZ treatment with auranofin in recurrent glioblastoma (NCT02770378). However, the exact role of the Trx system in GBM and more specifically in chemoresistance is still not clear at present.

Here, we present data confirming a substantial impact of the Trx/TXNIP system on intrinsic chemoresistance of GBM cells to cytostatic drugs which could pave the way to alternative treatment modalities.

## 2. Results

### 2.1. Trx-Inhibition Triggers Apoptosis in Human GBM Cell Lines

**Figure 1:**
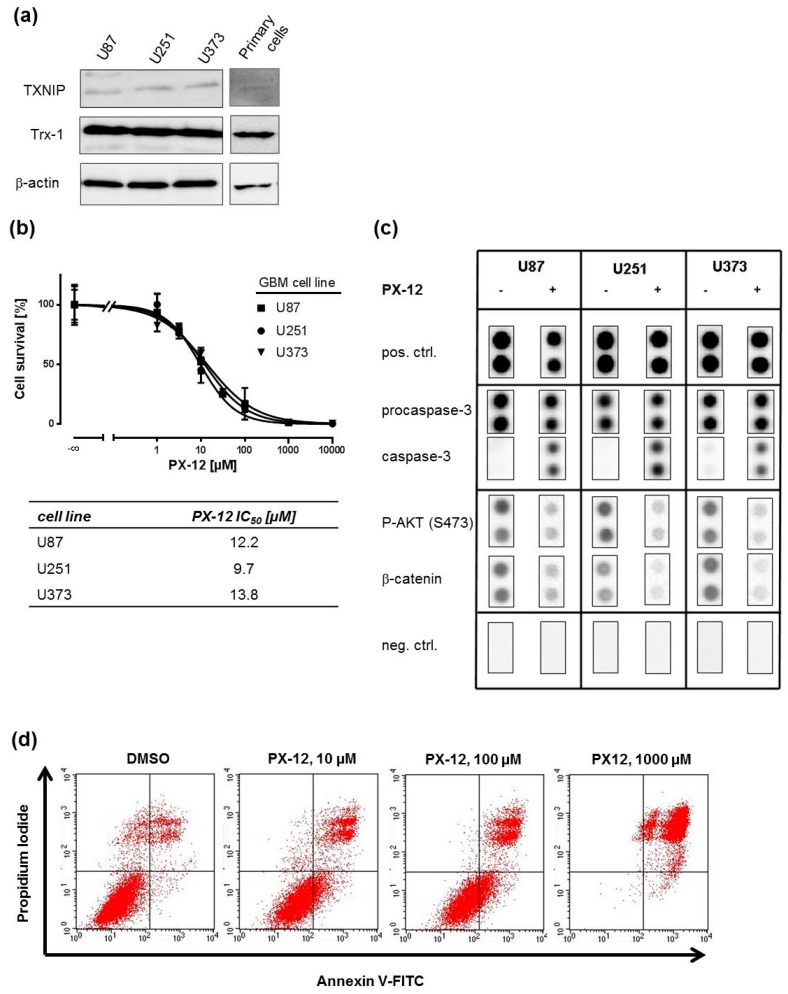
Pharmacological inhibition of thioredoxin (Trx) increases cell death via induction of apoptosis in human glioblastoma multiforme (GBM) cell lines. (**a**) Representative Western blots showing endogenous Trx-1 and Trx-interacting protein (TXNIP) expression in U87, U251, U373, and primary GBM cells. β-actin Western blot was performed to control for loading; (**b**) 3-(4,5-dimethylthiazol-2-yl)-2,5-diphenyltetrazolium bromide (MTT) concentration-response curves after 1-methyl-2-imidazolyl-disulfide (PX-12) exposure for 72 h in U87, U251, and U373 cells (upper graph). Respective PX-12 IC_50_ values are displayed in the table below the graph (*n* = 4); (**c**) Human proteome-profiler antibody arrays were used to assess the expression/phosphorylation of AKT (S473), procaspase-3, caspase-3, and β-catenin 72 h after PX-12 exposure; (**d**) Representative FACS blots of U251 cells treated with either vehicle (DMSO) or increasing PX-12 concentrations for 72 h as indicated. Annexin V positive cells were regarded as apoptotic cells.

GBM cell lines and high-grade glioma tissue have been reported to express significant amounts of Trx [16,28,29]. In order to confirm this, we determined Trx and TXNIP expression in three established GBM cell lines—U87, U251, and U373—and primary GBM cells, which have previously been shown by us to be intrinsically resistant to TMZ and cisplatin [30]. Trx-1 and TXNIP protein was expressed in all cells as determined by Western blotting (Figure 1a). To investigate whether GBM cell lines are responsive to Trx-inhibition, 1-methyl-2-imidazolyl-disulfide (PX-12), a pharmacological inhibitor of Trx, was applied. First, we investigated the effect of PX-12 at increasing concentrations on cell survival in 3-(4,5-dimethylthiazol-2-yl)-2,5-diphenyltetrazolium bromide (MTT) assays (Figure 1b, upper graph). An IC_50_ concentration of around 10 µM was determined for all three cell lines (Figure 1b, lower table). This concentration was used to assess the effect of PX-12 on apoptosis-related proteins such as (pro-)caspase-3, AKT-kinase (protein kinase B), and β-catenin (Figure 1c). PX-12 strongly induced expression of active caspase-3 and reduced phosphorylation of AKT-kinase and expression of β-catenin in a similar fashion in all three cell lines. These findings are in line with a concentration-dependent increase of apoptotic rates of U251 cells after PX-12 exposure as determined by annexin-V/propidium iodide staining (Figure 1d). Similar results were obtained with U87 and U373 cells (Figure S1).

### 2.2. Pharmacological Trx Inhibition Sensitizes Human GBM Cells to Cytostatic Drugs

**Figure 2:**
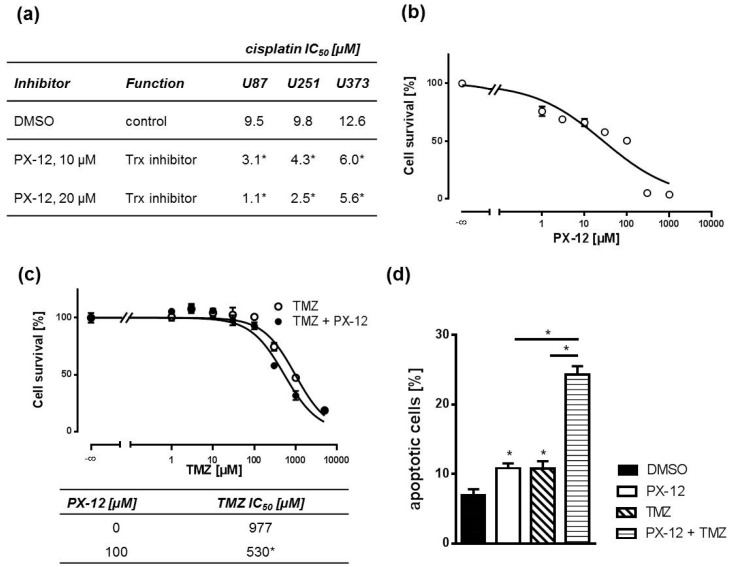
PX-12 treatment sensitizes human GBM cells to cytostatic drug treatment. (**a**) PX-12 causes a concentration-dependent reversal of cisplatin resistance. IC_50_ concentrations of cisplatin in the absence and presence of 10 µM and 20 µM PX-12 for 72 h, respectively, were obtained from sigmoidal concentration-response curves (0.03–300 µM cisplatin) of MTT assays (*n* = 3, * *p* < 0.05); (**b**) MTT assays with a concentration-response curve of PX-12 in primary human GBM cells treated for 72 h (*n* = 3); (**c**) Temozolomide (TMZ) concentration-response curves showing sensitization of primary GBM cells at 100 µM PX-12 incubated for 72 h (upper graph). Respective TMZ IC_50_ values are depicted in the table below the graph (*n* = 3, * *p* < 0.05); (**d**) Apoptotic rates as determined by annexin-V assays of primary GBM cells treated with either vehicle (DMSO), TMZ (1000 µM), PX-12 (100 µM), or a combination of both for 48 h as indicated. Annexin V positive cells were regarded as apoptotic cells (*n* = 3, * *p* < 0.05).

Next, we investigated whether Trx inhibition by PX-12 sensitizes GBM cell lines to the cytostatic drug cisplatin in MTT assays. Yet, TMZ is the only substance for which a statistically significant and clinically relevant benefit in both overall survival and progression-free survival has been demonstrated [4]. However, it is very difficult to handle in the laboratory setting as it must be given to GBM cells in concentrations as high as or even beyond its solubility [30]. Thus, we also used cisplatin as a model substance with a similar molecular mechanism of action: both substances interfere with DNA, resulting in strand breaks and apoptosis. Therefore, we treated cells with an IC_50_ concentration of PX-12 and increasing concentrations of cisplatin. Treatment with 10 µM PX-12 sensitized all GBM cell lines significantly to cisplatin when administered 1 h prior to cisplatin exposure. The sensitizing effect was concentration dependent, since 20 µM PX-12 triggered a more pronounced effect (Figure 2a). Next, we asked if our findings can be translated to a more clinically relevant scenario of primary GBM cells derived from a tumor biopsy. Initially, we determined the IC_50_ concentration of PX-12 in these cells (Figure 2b). Thereafter, we treated primary cells with this IC_50_ concentration and increasing TMZ concentrations. As observed with cisplatin in GBM cell lines, PX-12 was also capable of sensitizing primary GBM cells to TMZ as assessed by MTT assays (Figure 2c). This was paralleled by an increase in apoptotic rates after concomitant treatment of IC_50_ concentrations of PX-12 and TMZ as compared to single treatment alone (Figure 2d and Figure S2).

### 2.3. TXNIP Overexpression Chemosensitizes Human GBM Cell Lines

**Figure 3:**
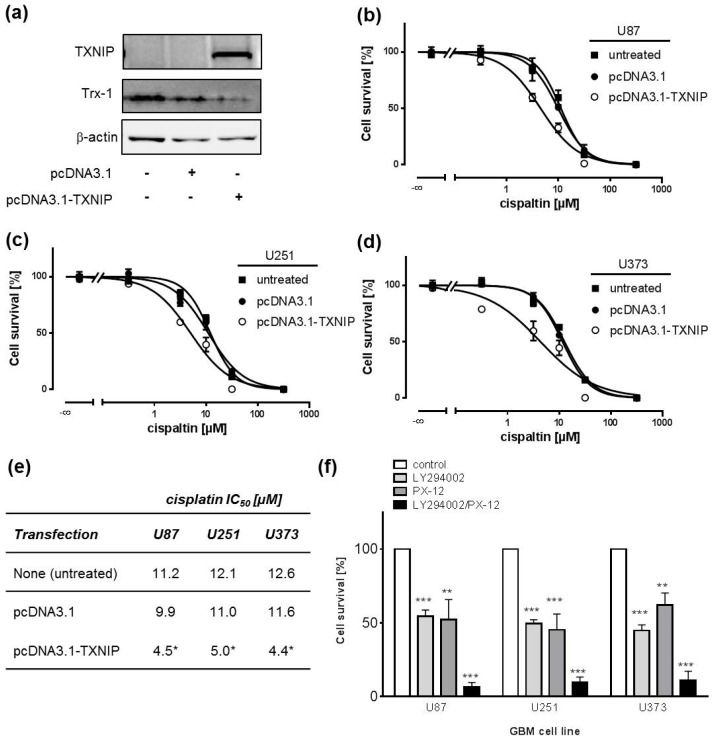
Overexpression of the tumour suppressor TXNIP inhibits Trx expression and sensitizes GBM cell lines to cisplatin. (**a**) Representative Western blots of U87 cells showing overexpression of TXNIP and downregulation of Trx-1 protein expression after transient transfection with pcDNA3.1-TXNIP or empty vector (pcDNA3.1) 48 h post-transfection. β-actin Western blot was performed to control for loading; (**b**–**d**) MTT assays with cisplatin concentration-response curves showing sensitization of U87 (**b**), U251 (**c**), and U373 (**d**) cells after transient transfection with empty vector (pcDNA3.1) or pcDNA3.1-TXNIP. Cells were incubated with cisplatin for 72 h at 24 h post-transfection; (**e**) Respective cisplatin IC_50_ values derived from curves presented in **b**–**d** (*n* = 4, * *p* < 0.05); (**f**) Prevention of AKT kinase signaling and Trx deactivation triggers cell death in GBM cell lines. U87, U251, and U373 cells were treated with control (DMSO) or 5 µM of the phosphatidylinositide 3-kinase (PI3K) inhibitor LY294002, 10 µM PX-12, and a combination of both, respectively. After 72 h, cell viability was assessed by MTT assays (*n* = 3, ** *p* < 0.01, *** *p* < 0.001).

**Figure 4:**
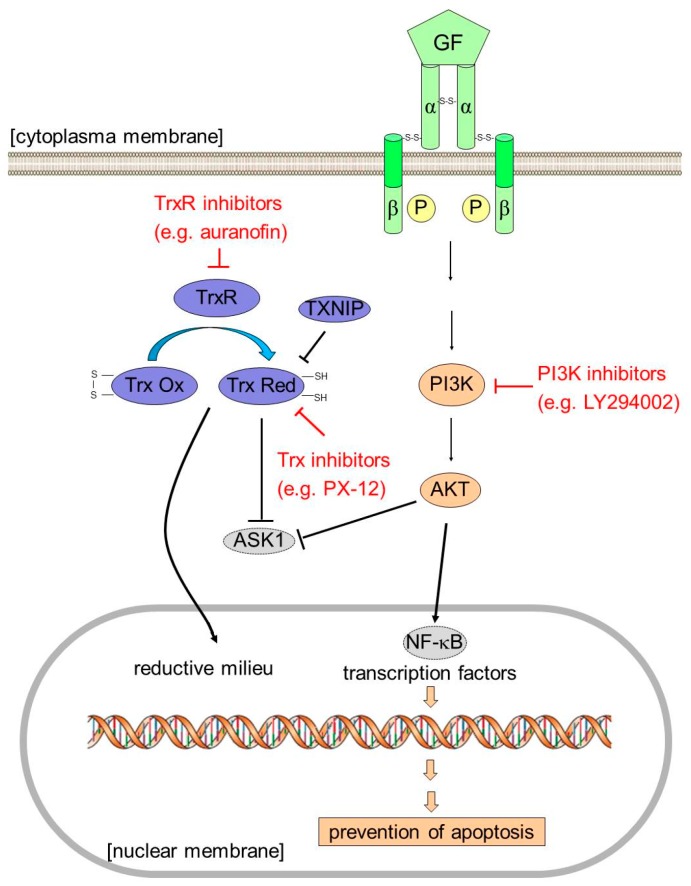
Schematic overview of the Trx system and tentative pathways involved in drug resistance of GBM cells. Potential drug insult to overcome resistance is presented in red. Abbreviations: AKT, protein kinase B (PKB); ASK1, apoptosis signal-regulating kinase 1; GF, growth factor; NF-κB, nuclear factor kappa-light-chain-enhancer of activated B cells; PI3K, phosphatidylinositide 3-kinase; Trx, thioredoxin; TrxR, Trx-reductase TXNIP, Trx-interacting protein.

In order to confirm our data, we overexpressed TXNIP in GBM cell lines as a genetic tool to inhibit Trx function and expression. We were not able to achieve sufficient expression of TXNIP in primary GBM cells after transfection. Therefore, we focused our experiments with TXNIP on GBM cells lines. Trx-1 expression was nearly abrogated after *TXNIP* transfection as exemplarily depicted for U87 cells (Figure 3a). Overexpression of TXNIP and consequently reduced Trx-1 expression significantly sensitized cells towards cisplatin when compared to empty vector transfected cells (Figure 3b–e). It has previously been shown by us and others that pharmacological inhibition of phosphatidylinositide 3-kinase (PI3K) sensitizes GBM cells to cisplatin and TMZ [30]. Reports from the literature indicate that PI3K/AKT signaling and the Trx system can be interconnected on various levels [31]. Therefore, we sought to combine both mechanisms and measure the impact on cisplatin resistance status of GBM cell lines. However, combining PI3K inhibition with anti-Trx treatment did not yield a window large enough to record a cisplatin concentration-response curve. Thus, we assessed the impact of the PI3K inhibitor LY294002 and PX-12 and a combination of both on cell survival in GBM cells. Both substances show an additive behavior in MTT assays (Figure 3f). We conclude that both mechanisms are closely interconnected (Figure 4).

## 3. Discussion

Despite its low incidence of about 30,000 new diagnoses per year, GBM is one of the deadliest cancer types, with a 5-year survival of 6% [1,5]. Drug resistance, radio resistance, and inoperability result in a useless medical armamentarium. The only improvement in GBM therapy, which leads to prolonged overall survival, is adjuvant administration of TMZ following radiotherapy [4]. Here, we present a mechanism of how resistance to cytostatic drugs is brought about in GBM cells. The mechanism we discovered operates on Trx overexpression. High Trx levels can be regarded as a marker for increased tumor aggressiveness in general [18]. In addition, in glioma downregulation of the negative Trx regulator TXNIP has been shown to be associated with higher histopathological glioma grade and shorter overall patient survival [15]. In line with that, Trx expression increases with glioma grade [16]. Trx overexpression was demonstrated to provoke vascular endothelial growth factor (VEGF) secretion and angiogenesis [32]. In invasive cervical carcinomas, Trx upregulation was detected in tumor microregions suffering from hypoxia, suggesting hypoxia-inducible factor-1 α (HIF-1α) induction, which is entailed by PI3K/AKT pathway activation [33]. Consistently, hypoxia was also linked to increased Trx levels and sensitivity to TrxR inhibition in cancer cells cultured in monolayers and 3D spheroid-based culture models [34]. Additionally, *TRX* gene transfection of lymphoid and breast cancer cells or Trx protein supplemented to tissue culture medium has been reported to prevent apoptosis and increase cell invasion [11,17]. With respect to anticancer drug resistance, *TRX* transfected murine WEHI7.2 lymphoma cells show significantly lower apoptosis rates when exposed to the anticancer drug etoposide, a topoisomerase-II inhibitor [35]. In this context, it is noteworthy that the TrxR inhibitor auranofin is currently evaluated in clinical trials as an anticancer agent alone or in combination with other drugs (NCT03456700, NCT01737502, NCT01747798, NCT02770378). There is strong evidence that the cytostatic activity of auranofin and other TrxR inhibitors on various cancer cells is mediated via an increase in cellular ROS, subsequently leading to apoptosis [17,19,20,21,22,26]. Other mechanisms discussed are inhibition of the PI3K/AKT pathway in lung cancer cells [23,36], inhibition of the proteasome in rhabdomyosarcoma and hepatoma cells [25,37], and adenosine triphosphate (ATP) depletion by inhibition of glycolysis in stem-like cancer cells [24]. The sensitivity of gastric cancer cells to auranofin could be increased by the silencing of genes involved in autophagy [38], and *BRCA1* deficiency has further been implicated to increase sensitivity of ovarian cancer cells to auranofin [39]. Synergistic activity on cancer cell growth in preclinical models is reported for the combination of auranofin with many anticancer agents and is in line with the use of auranofin as combination therapy in current clinical trials [24,25,36,40,41]. The sensitizing activity of auranofin is further supported by studies showing an enhancement of radiation response in breast cancer cells [42,43]. Furthermore, auranofin treatment has been described to overcome cisplatin resistance in ovarian cancer cells [27].

Thus, with respect to tailored medicine, Trx and TrxR are promising markers and emerging drug targets—if not alone, then at least in combination with cytostatic drugs or radiation as a chemosensitizer. In our study we confirmed Trx-1 and TXNIP expression in three GBM cell lines and primary cells. It has previously been shown by us that these cell lines are intrinsically resistant to TMZ and cisplatin [30]. This further drove us to investigate the role of Trx in chemoresistance. To this end, we applied PX-12, a small-molecule inhibitor which has already been demonstrated to overcome drug resistance in multiple myeloma [44,45], alone and in combination with cisplatin or TMZ. PX-12 chemically belongs to the group of alkylated 2-imidazolyl disulfide Trx inhibitors. On the molecular level, it covalently binds Cys-73 of Trx, thus thioalkylating the protein [46].

PX-12 treatment, just like genetic TXNIP overexpression, restored sensitivity to cytostatic drug treatment. Thus, we present a new treatment modality to overcome Trx-mediated ineffectiveness of drug therapy in GBM.

It is noteworthy that we and others have demonstrated that PI3K inhibition sensitizes GBM cells to alkylating drugs [30]. Reports from the literature provide evidence of a crosstalk of PI3K/AKT and the Trx system on various levels depending on the cell type [31]. Trx was observed to inhibit PTEN lipid phosphatase activity, thereby leading to unbraked PI3K-stimulated AKT-kinase activation [47]. This is underlined by the fact that TrxR inhibition by auranofin inhibited the PI3K/AKT pathway in lung cancer cells [23,36]. However, this mechanism might not operate in GBM, since all cell lines under investigation carry *PTEN* mutations. Apart from that, it is known that AKT-kinase functions in concert with Trx upstream of nuclear factor kappa-light-chain-enhancer of activated B cells (NF-κB). In this doublet, Trx preserves a reductive nuclear condition, which is a prerequisite for the transcription factor to bind DNA [48]. To this end, it is noteworthy that NF-κB is overexpressed frequently in GBM and is associated with chemoresistance [49]. Finally, Trx binds and inactivates apoptosis signal-regulating kinase 1 (ASK1). ASK1 is released from Trx binding upon oxidative stress to interact directly with AKT-kinase [50], which negatively regulates ASK1 activity by phosphorylation [51]. Consequently, oxidation or inactivation of Trx and reduced AKT activity lead to ASK1 activation, which in turn activates c-Jun N-terminal kinase (JNK) and p38 mitogen-activated protein (MAP) kinase pathways resulting in apoptosis [52].

Thus, we investigated whether both mechanisms are interconnected in GBM. If a sequential cascade would lead from Trx overexpression to AKT-kinase activation, inhibition of both principles should act in an additive manner. This would clear the path to so-called sequential therapy, a treatment modality where two sequential steps in a certain cascade are targeted simultaneously to ultimately block the pathway. In the context of this report, treatment of GBM cell lines with both a PI3K inhibitor and a Trx inhibitor should yield a high sensitivity to cytostatic drugs. However, we did not yield a window large enough to record a cisplatin concentration-response curve. However, cell survival assays under either inhibition alone and in combination revealed an additive interaction of both approaches in triggering cell death. Additionally, AKT-kinase phosphorylation and β-catenin protein expression were substantially reduced under the action of the Trx blocker PX-12, which also suggests that the AKT-kinase pathway is closely interconnected with Trx. We conclude that both principles act as a sequential cascade and could potentially present a new drug target (Figure 4). Combination of cytostatic drugs with Trx and/or PI3K inhibitors might thus result in clinical benefit for glioma patients. However, it has to be taken into account that the ability to cross the blood brain barrier is not common to all PI3K inhibitors and only a handful have entered into clinical trials so far [53]. No information could be retrieved regarding the ability of PX-12 to penetrate into the brain, leaving the question if PX-12 is a feasible option for glioma treatment.

## 4. Materials and Methods

### 4.1. Cell Culture, Transfection, and Preparation of Cell Lysates

All chemicals and cell lines—U87-MG, U251-MG, and U373-MG (Uppsala)—were obtained from Sigma-Aldrich (St. Louis, MO, USA), (HPA Culture Collections). Cell lines were not used beyond passage 20. U251 cells were cultivated in Roswell Park Memorial Institute (RPMI) 1640 Medium (Biochrom, Berlin, Germany) containing 10% fetal calf serum (FCS; Biochrom, Berlin, Germany), 100 IU/mL penicillin, and 100 µg/mL streptomycin (Biowest, Nuaillé, France) at 37 °C and 5% CO_2_. All other cell lines were cultivated in Dulbecco’s Modified Eagle Medium (DMEM) low Glucose (Biowest, Nuaillé, France) containing 10% FCS, 100 IU/mL penicillin, and 100 µg/mL streptomycin. Primary GBM cells were obtained from the University Hospital Cologne from a tumor biopsy of one patient with primary GBM (grade 4) according to the 2007 WHO classification [54]. The collection of tissue samples was approved by the University’s Institutional Ethical Board (project number: 03-170, approve date: 23.04.2012). Informed consent of the patient was obtained according to the Helsinki declaration of ethical requirements. Tissue specimens were obtained during neurosurgery, directly put on DMEM (+glucose, +glutamine, +pyruvate) on ice in the operation theatre, and immediately transported to the lab for further processing under sterile conditions. Specimens were cut into approximately 1-mm^3^ pieces with a scalpel and separately placed into 25-mm^2^ culture flasks with DMEM containing 10% FCS, 100 IU/mL penicillin, and 100 µg/mL streptomycin. Pieces were cultured for about 2–3 weeks at 37 °C, 5% CO_2_ until cells reached approx. 80% confluence. Thereafter, remaining tissue and debris was removed and only adherent cells were transferred into 75-mm^2^ culture flasks (passage 1), expanded again, and frozen (passage 2). For experiments, cells were thawed, expanded, and split for experiments (passage 3).

Full-length human TXNIP cDNA was subcloned in the mammalian expression vector pcDNA3.1 (Invitrogen, Carlsbad, CA, USA). Transient transfections were performed using Lipofectamin^®^2000 (Invitrogen, Carlsbad, CA, USA) according to the manufacturer’s instructions. Cells were grown to 70–90% confluence and transfected with a mixture of 3.5 µg (6-well) or 1 µg (24-well) plasmid, Lipofectamine^®^2000, and Opti-MEM^®^ Medium (Invitrogen, Carlsbad, CA, USA). Whole cell lysates were isolated 48 h after transfection to confirm TXNIP and Trx-1 expression by Western blotting.

In order to prepare whole cell lysates, cells were washed with ice cold phosphate-buffered saline (PBS) and lysed with ice cold Denaturing Cell Extraction Buffer (FNN00091, Thermo Fisher Scientific, Waltham, MA, USA), incubated on ice for 30 min, and centrifuged for 15 min at 4 °C. The supernatant was used for protein content determination and subsequent immunoblotting.

### 4.2. Proteome Profiling

The functional status of the signaling pathways was measured by Proteome Profiler^®^ human apoptosis and human phosphokinase arrays (R&D Systems ARY003B and ARY009, respectively, Minneapolis, MN, USA). Only membranes of the same lot numbers were used for comparison. Cells were lysed after incubation with PX-12 by addition of ice-cold lysis buffer to PBS washed culture plates. After centrifugation, the protein content of the supernatant was determined and 300 µg of total protein was used for proteome profiling according to the manufacturer´s protocol and as described previously [55].

### 4.3. Western Blot Analysis

For immunoblotting, standard procedures were used as previously described [55]. Thirty micrograms of total protein were loaded onto Criterion^®^ Tris-HCL protein gels (Biorad, Hercules, CA, USA). After electrophoresis, gels were blotted onto polyvinylidene fluoride (PVDF) membranes (Merck Millipore, Burlington, MA, USA) and blocked with 5% milk/Tris-buffered saline-Tween (TBS-T). The following antibodies were used: Anti-Trx-1 (1:2000; AF 1970) combined with donkey anti-goat IgG-HRP (1:10,000; HAF 109; both R&D systems, Minneapolis, MN, USA), anti-VDUP1 (1:500; D2, Santa Cruz Biotechnology, Dallas, TX, USA) combined with goat anti-mouse IgG (H + L)-HRP (1:10,000; Thermo Scientific, Waltham, MA, USA), and anti-β-actin-HRP (1:10,000; C-4, Santa Cruz Biotechnology, Dallas, TX, USA). Primary antibodies were applied in 1% milk/TBS-T at 4 °C overnight and secondary antibodies in 1% milk/TBS-T for 1 h. Immunoblots were developed with the enhanced chemoluminescence system (Amersham Biosciences, Little Chalfont, UK).

### 4.4. MTT Assay

Cell survival after exposure to cisplatin or TMZ and in the presence of signaling inhibitors was determined by MTT assays as previously described [55]. Briefly, 5000 (U251, U87, U373) or 15,000 (primary) cells were plated on 96 wells and grown at 37 °C and 5% CO_2_ overnight. Thereafter, signaling inhibitors PX-12 (1–10.000 µM) and LY294002 (5 µM) were added to culture media 1 h prior to addition of cisplatin or TMZ for 72 h as indicated. Final DMSO concentrations in media did not exceed 1%. For the analysis of TXNIP overexpressing cells, 1 × 10^4^ cells were seeded into individual wells of a 24-well plate and transfected with Lipofectamine^®^2000 as described above. After 24 h, cells were exposed to increasing concentrations of cisplatin for 72 h.

### 4.5. Annexin V Apoptosis Assay

For apoptosis measurements, the BD Pharmingen^®^ FITC Annexin V Apoptosis Detection Kit (BD Biosciences, Franklin Lakes, NJ, USA) was used according to the manufacturer’s protocol. Briefly, 2.5 × 10^5^ cells were seeded into six-well plates and incubated at 37 °C and 5% CO_2_ overnight. After compound treatment for 48–72 h, cells were trypsinized and centrifuged for 4 min at 1500× *g*. Supernatant was removed and cells were resuspended in 500 µL binding buffer. Five microliters of propidium iodide and 5 µL Annexin V-FITC were mixed with 100 µL of cells in binding buffer. After 15 min of incubation on ice, samples were analyzed by flow cytometry (FACSCalibur^®^, BD Bioscience Franklin Lakes, NJ, USA).

### 4.6. Data Analysis and Statistical Methods

Concentration effect curves were fitted to data points by nonlinear regression analysis using the four-parameter logistic equation (GraphPad^®^ Prism, https://www.graphpad.com/scientific-software/prism/). Statistical differences between two groups were determined by unpaired two-tailed Student’s *t*-test. Comparisons among several groups were performed by ANOVA followed by Tukey’s post hoc test. Data are presented as means ± SEM.

## Supplementary Materials

Supplementary materials can be found at http://www.mdpi.com/1422-0067/19/10/2874/s1.

## Abbreviations

AKT	protein kinase B (PKB)
ATP	adenosine triphosphate
ASK1	apoptosis signal-regulating kinase 1
GBM	glioblastoma multiforme
GF	growth factor
HIF-1 α	hypoxia-inducible factor-1 α
JNK	c-Jun N-terminal kinase
MAP kinase	mitogen-activated protein kinase
PI3K	phosphatidylinositide 3-kinase
PTEN	phosphatase and tensin homolog
ROS	reactive oxygen species
TMZ	temozolomide
Trx	thioredoxin
TrxR	thioredoxin reductase
TXNIP	Trx-interacting protein
VDUP1	vitamin-D_3_-upregulated protein 1
VEGF	vascular endothelial growth factor
