# Supplementary material for: Thioredoxin Confers Intrinsic Resistance to Cytostatic Drugs in Human Glioma Cells
Source: Int J Mol Sci. 2018 Sep 21;19(10):2874. doi: 10.3390/ijms19102874 (PMC6212897; doi:10.3390/ijms19102874)
Supplement: Supplementary file 1 [file ijms-19-02874-s001.pdf]

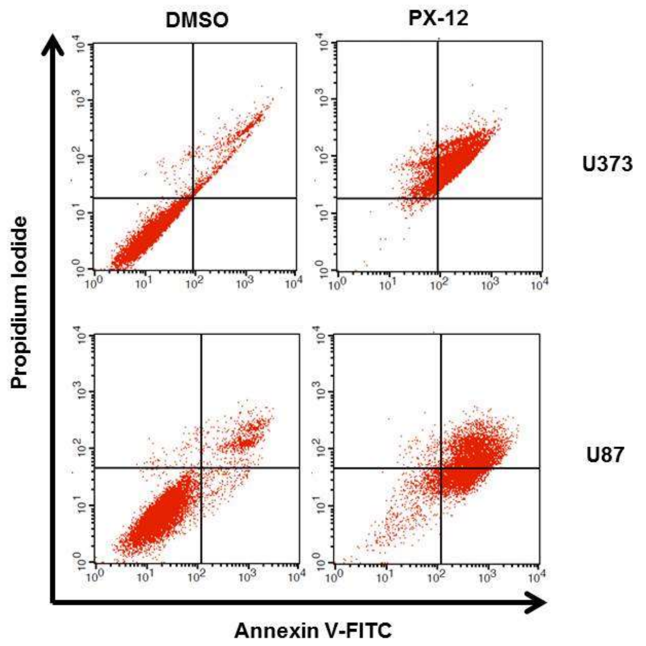

Figure S1. Representative FACS blots of U373 and U87 cells treated with either vehicle (DMSO) or 1000 μM PX-12 for 72 hours as indicated. Annexin V positive cells were regarded as apoptotic cells.

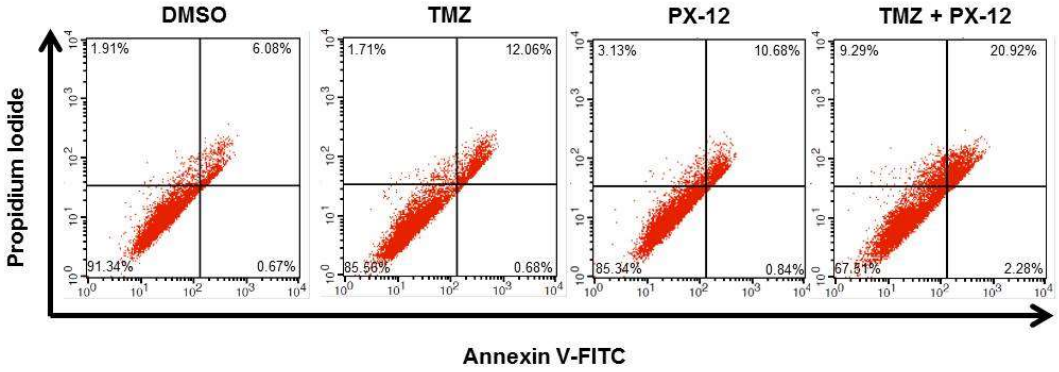

Figure S2. Representative FACS blots of primary GBM cells treated with either vehicle (DMSO) or 100 μM PX-12, 1000 μM TMZ, or a combination of both for 48 hours as indicated. Annexin V positive cells were regarded as apoptotic cells.
